# Rise in childhood obesity with persistently high rates of undernutrition among urban school-aged Indo-Asian children

**DOI:** 10.1136/adc.2007.125641

**Published:** 2007-10-17

**Authors:** T H Jafar, Z Qadri, M Islam, J Hatcher, Z A Bhutta, N Chaturvedi

**Affiliations:** 1 Clinical Epidemiology Unit, Department of Community Health Sciences, Aga Khan University, Karachi, Pakistan; 2 Section of Nephrology, Department of Medicine, Aga Khan University, Karachi, Pakistan; 3 Division of Nephrology, Department of Medicine, Tufts University School of Medicine, Boston, Massachusetts, USA; 4 National Heart and Lung Institute, Imperial College London, UK; 5 Department of Paediatrics and Child Health, Aga Khan University, Karachi, Pakistan

## Abstract

**Background:**

Childhood obesity is an emerging global public health challenge. Evidence for the transition in nutrition in Indo-Asian developing countries is lacking. We conducted these analyses to determine the trends in nutritional status of school-aged children in urban Pakistan.

**Methods:**

Data on the nutritional status of children aged 5 to 14 years from two independent population-based representative surveys, the urban component of the National Health Survey of Pakistan (NHSP; 1990–1994) and the Karachi survey (2004–2005), were analysed. Using normative data from children in the United States as the reference, trends for age- and gender-standardised prevalence (95% CI) of underweight (more than 2 SD below the weight-for-age reference), stunted (more than 2 SD below the height-for-age reference) and overweight and obese (body mass index (BMI) 85^th^ percentile or greater) children were compared for the two surveys. The association between physical activity and being overweight or obese was analysed in the Karachi survey using logistical regression analysis.

**Results:**

2074 children were included in the urban NHSP and 1675 in the Karachi survey. The prevalence of underweight children was 29.7% versus 27.3% (p = 0.12), stunting was 16.7% versus 14.3% (p = 0.05), and prevalence of overweight and obese children was 3.0 versus 5.7 (p<0.001) in the NHSP and Karachi surveys, respectively. Physical activity was inversely correlated with being overweight or obese (odds ratio, 95% CI, 0.51, 0.32–0.80 for those who engaged in more than 30 minutes of physical activity versus those engaged in less than 30 minutes’ activity).

**Conclusions:**

Our study highlights the challenge faced by Pakistani school-aged children. There has been a rapid rise in the number of overweight and obsese children despite a persistently high burden of undernutrition. Focus on prevention of obesity in children must include strategies for promoting physical activity.

The rising number of overweight and obese children and adolescents has been well documented in many developed countries.^1^ ^2^ Several studies have provided robust evidence that being overweight in childhood increases the risk of atherosclerosis, a risk that continues in to adulthood.^3^ This problem of atherosclerosis might be compounded in Indo-Asian children; muscle-thin, fat-rich babies have been shown to be particularly susceptible to insulin resistance in the presence of accelerated growth during childhood.^4^ ^5^

Traditionally, a deficiency in macro- and micronutrients has been the major problem among children in low-income countries.^6^^–^10 Nevertheless, owing to progressive urbanisation and the associated changes in lifestyle, the energy balance is shifting.^11^ Childhood obesity is becoming an equally challenging, yet under-recognised, problem in many emerging countries.^12^ Data on nutritional transitioning in children from low-income developing countries including those in Indo-Asia are scarce, however.

Previously we reported results from the NHSP showing that a quarter of the population aged 15 years or over was overweight or obese using Asian-specific thresholds.^13^ In this paper we report the trends in nutritional status for children aged 5 to 14 years obtained from two surveys in Pakistan, the NHSP (conducted during 1990–1994) and the Karachi Survey (2004–2005). In addition, data on physical activity and healthy dietary habits were also collected in the Karachi survey. Since each of these is an important determinant of weight and obesity, we also describe the relationship between activity and diet in urban Indo-Asian children.^14^ ^15^

## METHODS

### National Health Survey of Pakistan

Cross-sectional data were collected during the NHSP over four years (1990–1994) by the Pakistan Medical Research Council (PMRC) under the technical guidance and support of the US National Center for Health Statistics (NCHS). The overall design of the survey was a modification of the Third National Health and Nutrition Examination Survey (NHANES III) conducted by the NCHS, tailored to the needs of Pakistan. The details of the sampling, design, components, survey instrument and quality control have been previously reported.^16^ Briefly, the survey was conducted on a nationally representative sample of 18 135 individuals aged 6 months to 110 years from 2400 urban and rural households after obtaining informed consent. A two-stage stratified design was used. The urban and rural areas of each of the four provinces of Pakistan were taken as strata. There were 80 urban or rural primary sampling units. A total of 30 households were drawn into the sample from each unit, and all residents of the household were included in the study. The overall individual response rate was 92.6%. The total number of individuals aged 5 years or above was 15 083. Out of these, subjects aged under 15 years were classified as children (n = 5641) in the NHSP.

Data on demographic, lifestyle, socioeconomic and health-related variables were collected using a questionnaire validated in local languages. Physicians at mobile examination centres performed a standardised physical examination. Mothers were proxy respondents for children aged under 12 years. Trained technicians performed anthropometric examinations.

Weight and height were recorded to the nearest 0.1 kg and 0.1 cm, respectively, for each child in light clothing without shoes. The BMI was calculated as weight in kilograms divided by height in metres squared. Quality control for the survey included a visit to the field by expert consultants, duplicate examination by field supervisers, calibration protocols and retraining exercises.^16^

Urban and rural areas were classified according to the definition used by Federal Bureau of Statistics, where thinly populated, largely agricultural areas (fewer than 5000 people) with poor availability of basic amenities (eg, sewerage system, telephone lines) are considered rural.^13^

### Karachi survey

This survey was conducted in the urban city of Karachi, Pakistan, during 2004–2005 as part of the baseline data for the trial of a community intervention for the control of hypertension. Ethical approval was obtained from the Aga Khan University Ethics Review Committee.

Twelve geographical clusters were randomly chosen from among the 72 low-to-middle-income clusters in Karachi. There were approximately 250 households in each cluster. In each household with children between the ages of 5 to 14 years, one child was randomly chosen, and informed consent was obtained from the parent and child for those over the age of 9. A total of 1675 children between the ages of 5–14 years were enrolled in the study.

Information was collected for each child on their age, gender and educational status. Children under 9 years of age were interviewed in the presence of their parent or guardian. Information on physical activities was collected through questions related to the amount of time in the past 7 days the child had spent in organised and other strenuous physical activity at school, if the child was at school during that period, and the amount of time they spent in similar activities out of school. Diet was measured by a limited food-frequency questionnaire that measured the number of servings of fruits and vegetables a child ate per day and whether they ate sweets. Weight and height measurements were obtained in the same way as in the NHSP (described above).

### Statistical analysis

Anthropometric indicators of nutritional status were used. Underweight was defined as having a weight-for-age below −2 SD from the World Health Organization/National Centre of Health Statistics (WHO/NCHS) reference, and stunting as having a height-for-age below −2 SD from the WHO/NCHS reference. Overweight and obese were defined as a BMI-for-age at the 85th percentile or greater from the 2000 Center for Disease Control growth charts^17^ ^18^ The overall and age-specific prevalence of nutritional status was computed in the urban and rural populations of those who participated in the NHSP and in the Karachi survey. All results were standardised for age and gender according to the NHSP.

We also performed a sensitivity analysis for the overall estimates of overweight or obese children using the International Obesity Task Force (IOTF) standard, equivalent to a BMI>25 kg/m^2^.^19^

The relationship between physical activity and being overweight or obese was assessed through logistical regression analysis using proc surveyfreq. SAS version 8.0. Survey design methods (clustering) were accounted for in all analyses.

## RESULTS

### NHSP 1990–1994

In the NHSP data, a total of 5641 subjects were aged between 5 to 14 years. 2074 children surveyed for NHSP were from urban areas. Of these, data on height and weight were available for 1972 children (95.0%), 1023 (51.9%) boys and 949 (48.1%) girls. Their mean age (SD) was 9.1 (2.8) years and their mean BMI was 15.0 (2.6) kg/m^2^.

In the urban areas, 29.7% (27.7–31.7%) of children were underweight: 32% boys and 28% girls, and 16.7% (15.1–18.3%) had stunted growth (both boys and girls). The prevalence of both being underweight and having stunted growth remained fairly constant until the age of 11–12 years old, but decreased for those aged 13–14 years old from 19% to 15% overall (table 1). Boys of all ages tended to have a higher prevalence of low weight and stunting. The overall prevalence of being overweight or obese was about 3.0% (2.2–3.8%) among both boys and girls. This prevalence increased with age, reaching 4% and 5% by 13–14 years in boys and girls, respectively.

**Table 1:** Age-specific nutritional status of urban school-aged children in the NHSP 1990–1994

Age (in years)	All			Boys					Girls			
	(Underweight) −2 SD below weight for age % (n)	(Stunted) −2 SD below height for age % (n)	(Overweight or obese) > 85^th ^percentile BMI for age % (n)		(Underweight) −2 SD below weight for age n (%)	(Stunted) −2 SD below height for age n (%)	(Overweight or obese) greater than 85^th^ percentile BMI for age n (%)	(Underweight) −2 SD below weight for age n (%)		(Stunted) −2 SD below height for age n (%)	(Overweight or obese) > 85^th^ perentile BMI for age n (%)	
5–6n = 452	30.3 (137)	16.2 (73)	3.8 (17)		32.5 (80)	17.1 (42)	3.3 (08)	27.7 (57)		15.0 (31)	4.4 (09)	
7–8n = 468	31.2 (146)	19.2 (90)	1.5 (07)		34.1 (79)	17.7 (41)	2.2 (05)	28.4 (67)		20.8 (49)	0.8 (02)	
9–10n = 367	31.3 (115)	16.9 (62)	2.2 (08)		33.8 (66)	19.0 (37)	2.1 (04)	28.5 (49)		14.5 (25)	2.3 (04)	
11–12 n = 383	34.2 (131)	15.9 (61)	4.2 (16)		32.2 (65)	16.3 (33)	4.0 (08)	36.5 (66)		15.5 (28)	4.4 (08)	
13–14 n = 302	18.2 (56)	14.6 (44)	4.0 (12)		21.6 (32)	12.8 (19)	3.4 (05)	15.6 (24)		16.2 (25)	4.5 (07)	
**5–14 n = 1972**	**29.7 (585)**	**16.7 (330)**	**3.0 (60)**		**31.5 (322)**	**16.8 (172)**	**2.9 (30)**	**27.7 (263)**		**16.6 (158)**	**3.2 (30)**	

Using IOTF standards, the prevalence of overweight or obese children in urban NHSP was 2.7%.

Overall, only 15% of children reported daily consumption of fruit: 11% in rural and 19% in urban areas, and 28% reported daily consumption of vegetables, both in rural and urban areas.

### Karachi survey

We interviewed 1675 children of whom 855 (51%) were boys and 820 (49%) were girls. Measurements of weight and height were recorded in all cases. The mean age (SD) of children was 9.4 (2.8) years, and the mean BMI was 15.4 (3.0) kg/m^2^.

#### Nutritional status in Karachi survey

About 27.9% (95% CI) (26–30%) of children were underweight: 29% boys versus 27% girls (p = 0.39), and 14.6% (13–16%) were stunted: 15.5% boys versus 13.8% girls (p = 0.36). The pattern with age was similar to that seen in the NHSP, remaining fairly stable until the age of 11–12 years (prevalence of 16%) and then decreasing with a prevalence of underweight and stunted children among those aged 13–14 years, respectively (prevalence of 9%) (table 2). The overall prevalence of overweight and obese children was 5.7% (4.4–6.0%): 4.6% boys versus 6.4% girls (p = 0.13). The number of overweight and obese children increased with age, reaching 7% and 11% in boys and girls aged 13–14 years, respectively.

**Table 2:** Age-specific and age-standardised nutritional status of urban school-aged children in the Karachi survey 2004–2005

Age (in years)	Overall			Boys			Girls		
	(Underweight) −2 SD below weight for age n (%)	(Stunted) −2 SD below height for age n (%)	(Overweight or obese) >85^th^ percentile BMI for age n (%)	(Underweight) −2 SD below weight for age n (%)	(Stunted) −2 SD below height for age n (%)	(Overweight or obese) >85^th^ percentile BMI for age n (%)	(Underweight) −2 SD below weight for age n (%)	(Stunted) −2 SD below height for age n (%)	(Overweight or obese) >85^th^ percentile BMI for age n (%)
5–6n = 334	30.9 (102)	14.3 (47)	4.4 (15)	30.0 (55)	14.5 (26)	3.7 (07)	32.2 (47)	14.1 (21)	5.4 (08)
7–8n = 371	29.2 (108)	17.8 (65)	3.8 (13)	31.7 (57)	18.2 (32)	3.2 (05)	25.9 (51)	17.5 (33)	4.4 (08)
9–10n = 333	30.5 (101)	16.0 (52)	5.8 (20)	29.9 (48)	20.3 (32)	4.1 (08)	31.1 (53)	11.9 (20)	7.1 (12)
11–12n = 343	28.7 (99)	14.4 (50)	6.0 (21)	29.6 (58)	13.7 (27)	5.6 (11)	27.4 (41)	15.2 (23)	6.6 (1.0)
13–14n = 294	16.1 (48)	8.9 (26)	9.1 (27)	21.4 (28)	9.8 (13)	7.4 (10)	13.1 (20)	8.9 (13)	10.5 (17)
5–14n = 1675	27.9 (458)	14.6 (240)	5.7 (96)	29.0 (246)	15.5 (130)	4.6 (41)	26.9 (212)	13.8 (110)	6.4 (55)

Using IOTF standards, the prevalence of overweight or obese children in the Karachi survey was 5%.

No child reportedly ate the recommended 4–5 daily servings of fruit and vegetables. Approximately one fifth of children (19.8%, 95% CI 12.9% to 26.6%) stated that they ate no fruits and vegetables. The majority of children ate chocolate or other sweets at least once a day (71.2%, 95% CI 66.2% to 76.2%).

**Figure 1:**
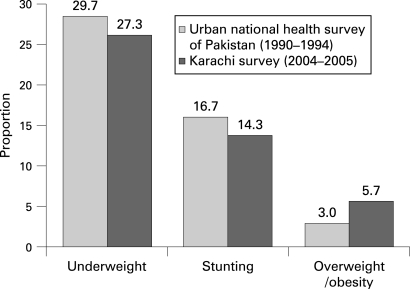
Trends in nutrition in urban Pakistan.

A comparison of nutritional trends between the two urban surveys is illustrated in figure 1. The overall prevalence of being underweight was 29.7% versus 27.3% (p = 0.12), stunting was 16.7% versus 14.3% (p = 0.05), and the prevalence of being overweight or obese was 3.0 versus 5.7 (p<0.001) in the NHSP versus the Karachi survey, respectively. Among boys, the prevalence of being underweight was 31.5% versus 29.0% (p = 0.26), stunting was 16.8% versus 15.5% (p = 0.45), and prevalence of being overweight or obese was 2.9% versus 4.6% (p = 0.06) in the NHSP versus the Karachi survey, respectively.

Among girls, the prevalence of being underweight was 27.7 % versus 26.9% (p = 0.75), stunting was 16.6% versus 13.8% (p = 0.12), and prevalence of being overweight and obese was 3.2% versus 6.4% (p = 0.002) in the NHSP versus the Karachi survey, respectively.

#### Physical activity in the Karachi survey

Data on physical activity were collected for 1669 children (table 3). The median daily amount of time when children were physically active in Karachi was just over half an hour, with boys being more than twice as active as girls (55 minutes (IQR 13, 120 minutes) versus 25 minutes (IQR 0,60 minutes) respectively). Over 20% of children (boys 16% (95% CI 13% to 20%) and girls 27% (95% CI 21% to 32%)) did no exercise either in or out of school.

**Table 3:** Total physical activity among children aged 5–14 years in Karachi

	Boys % (n)	Girls % (n)	Total % (n)
Formal in school	394	389	783
None	65.0 (256)	66.6 (259)	65.8 (515)
1–30 minutes	13.8 (133)	33.1 (129)	33.5 (262)
⩾ 31 minutes	1.3 (05)	0.3 (01)	0.8 (06)
Informal in school	394	389	783
None	31.5 (124)	38.1 (148)	34.7 (272)
1–30 minutes	58.9 (232)	56.8 (221)	57.9 (453)
31–60 minutes	6.6 (26)	4.9 (19)	5.7 (45)
>60 minutes	3.0 (12)	0.3 (01)	1.7 (13)
Out of school	854	815	1669
None	21.4 (183)	36.2 (295)	28.6 (478)
1–30 minutes	25.3 (216)	28.0 (228)	26.6 (444)
31–60 minutes	23.1 (197)	18.9 (154)	21.0 (351)
>60 minutes	30.2 (258)	16.9 (138)	23.7 (396)
Total	854	815	1669
None	16.2 (138)	26.5 (216)	21.2 (354)
1–30 minutes	22.7 (194)	29.7 (242)	26.1 (436)
31–60 minutes	21.4 (183)	19.4 (158)	20.4 (341)
>60 minutes	39.7 (339)	24.4 (199)	32.2 (538)

About 66% (95% CI 52% to 80%) of school-attending children reported participating in no organised sport within school, and for those that did, it was usually for 30 minutes or less per school day (33% (95% CI (20% to 47%) of children). Although 65% (95% CI 58% to 72%) of children reported other exercise in school, the majority (58% (95% CI 52% to 64%)) did less than 30 minutes of this type of activity.

Overall 29% (95% CI 25% to 36%) of children did no exercise out of school (boys 21% (95% CI 17% to 26%), girls 36% (95% CI 31% to 41%)), and only 24% (95% CI 20% to 28%) did more than 1 hour of out of school exercise (boys 30% (95% CI 25% to 36%), girls 17% (95% CI 14% to 20%)).

The amount of physical activity decreased with age, particularly after the age of 9 for girls and 11 for boys. The median time spent watching television was 120 minutes per day, interquartile range (60,180 minutes). There is little variation by age or gender, 5–6 year olds watched somewhat less television (median 60 minutes (IQR (30,120)), whereas girls aged 13 to 14 years watched somewhat more (median 150 minutes (IQR (90, 240 minutes)).

**Figure 2:**
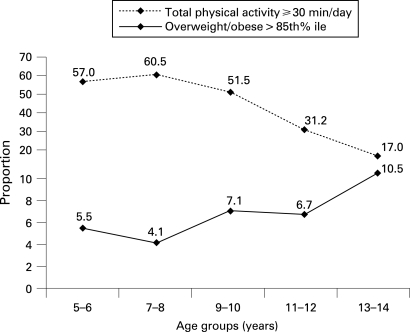
Age-related changes in total physical activity and prevalence of overweight and obese girls in Karachi.

The results of regression analyses revealed that those engaged in more than 30 minutes of physical activity had significantly lower odds (OR, 95% CI 0.51, 0.32–0.80) of being overweight or obese than those who participated in less than 30 minutes’ activity. The relationship between being overweight or obesity and physical activity at various age groups shows an inverse relationship between the amount of exercise and the prevalence of obesity, especially in girls (fig 2).

## DISCUSSION

This is the first report of the nutritional status of the Indo-Asian children of Pakistan, based on a sound, nationally representative epidemiological survey. It is also the first trend analysis in urban Pakistan. Using normative data from children in the United States as a reference, over a 10-year period in urban areas, there was a slight reduction in the prevalence of underweight children and those with stunting, and a marked increase in overweight and obese children 3.0 versus 5.7% (p<0.001). Physical activity was inversely correlated with being overweight or obese (odds ratio, 95% CI, 0.51, 0.32–0.80 for those engaged in 30 minutes or more physical activity compared with those who engaged in less that 30 minutes’ activity).

In both surveys, the prevalence of undernutrition decreased whereas the number of overweight or obese children increased with age. Our study therefore highlights the unique challenge faced by school-aged children in Pakistan: a rapid increase in the proportion of children with overnutrition in the presence of a persistently high burden of undernutrition.

The high prevalence of undernutrition in terms of macronutrient as well as micronutrient deficiencies is a well-recognised problem among children under 5 years of age in the Asian developing countries, ranging from 16.0% in the People’s Republic of China to 64.0% in Bangladesh.^6^ The etiologies of these conditions are multi-factorial and are often attributed to poor maternal nutritional, poor access to healthcare and low birth weight. Adequate feeding, however, can lead to catch-up growth, which negates some of these adverse influences.^20^^–^22 Our analyses show that a substantial proportion of the population is still underweight even during school years and adolescence in Pakistan. Moreover, the relatively static nature of these estimates from the recent Karachi survey indicates that no substantial improvement in nutritional status has been made. In part, this could be attributed to poor sanitation and the associated frequent diarrhea and an inability to properly digest food, which are consistent with other reports from Indo-Asia.^7^ ^23^ Our findings emphasise the need to implement evidence-based strategies for improving nutritional indicators in this region.^24^ ^25^

At the same time, our results highlight the alarmingly rapid rise in the number of overweight and obese school-aged children in urban Pakistan. An almost twofold increase was observed in the estimated levels over a decade, regardless of whether the WHO/NCHS (from 3% to 5.5%) or the IOTF (from 2.7% to 5.0%) criteria were used as a reference. These patterns are consistent with global trends in childhood obesity observed in developed countries such as the United States, where the prevalence of overweight and obese increased from 14% to 17% over 4 years (NHANES 1999–2000 and 2003–2004) and in other emerging countries such as Brazil where a 2.5-fold increase in overweight and obesity children (from 4 to 14%) over the past three decades has been reported.^12^ ^26^

The age-related rise in overweight and obese children in Karachi was associated with a parallel decline in physical activity, suggesting that alteration in the energy balance is one of the major contributing factors. We found that only 7% of girls and 30% of boys aged 13–14 years do the recommended physical activity of 1 hour per day.^27^ The benefits of increasing physical activity for reducing obesity as well as other health benefits are well established.^28^ ^29^ Thus, special efforts are needed to engage children, particularly girls, in sports and related activities as they approach puberty. These efforts will include policy-level changes that will help create safe environments for exercise.^30^ In addition, family-based, culturally relevant behavioural interventions that have been shown to be effective in weight management elsewhere need to be tested and implemented in developing-country settings.^31^ ^32^

At the same time, it must be kept in perspective that up to about 30% of the school-aged children still fall into the underweight category, although this proportion decreases with age (table 2). Moreover, micronutrient deficiencies also continue to be a challenge.^33^

The US Department of Health and Human services, and the American Heart Association, along with other similar organisations recommend at least five servings of fruits and/or vegetables per day.^34^ We found that the dietary habits of children were unhealthy in Karachi, with the vast majority consuming grossly inadequate amounts of fruits and vegetables. The consumption of fruits and vegetables continues to be inadequate in the United States and United Kingdom as well.^34^ ^35^ Thus, population-wide measures are needed worldwide for improving healthy dietary habits among children. Such measures should include ensuring the affordability of these choices. The promotion of physical activity in these children should also be encouraged.

Our study has limitations. First, the NCHS/WHO references for school-aged children and adolescents have a number of methodological and practical problems, which may be magnified in Indo-Asian populations. It is important to underscore that the definition for being overweight and obese in our study used references based on Western populations. Our analysis of the NHSP data in adults suggested lower BMI thresholds than those recommended for Western populations for defining being overweight and obese children on the basis of their association with chronic disease outcomes.^13^ These findings have been confirmed by others.^36^ This is thought to be due to greater central fat deposition and insulin resistance in Indo-Asians compared with Europeans, the risk of which probably begins in utero.^37^ Although our previous work has shown higher BMI-adjusted blood pressure in Pakistani children compared with white children in the United States, corresponding thresholds of Asian-specific BMI in children for defining who is overweight and obese are not available.^38^ There is a need to develop appropriate yardsticks for measuring these risks in Indo-Asian populations. Furthermore, this downward shift in the relationship between obesity and adverse outcomes would suggest lowering the current operational thresholds for defining being underweight and having stunted growth for this population as well.^39^ In that light, our analyses might have overestimated and underestimated the true burdens of undernutrition and overnutrition, respectively. Cohort studies of function and disease outcomes are needed to develop and validate appropriate thresholds in this population. However, our analyses provide robust evidence of the high magnitude of the double burden of this problem and, more importantly, of a steep rise in the percentage of overweight and obese children. Furthermore, it is becoming evident that an accelerated rate of weight gain in childhood, regardless of the threshold, is a powerful indicator of future risk of chronic disease.^37^ Disturbingly, our analyses show that this phenomenon is being experienced in urban Pakistan, and possibly in neighbouring countries.

Second, of the two surveys that were compared, the NHSP was nationally representative whereas the Karachi survey was representative of an urban city in the province of Sind. This is because follow-up data at a national level or even representative data from rural areas are not available. Only urban parts of the NHSP, however, were considered for trend analysis with the Karachi survey, and both the surveys employed similar multistage cluster-sampling strategies and measurement protocols. Moreover, no major differences in estimates of nutritional status among provinces were noted in urban NHSP (unpublished data). Therefore, we believe the surveys are comparable and that the findings of Karachi would be generalisable to other urban cities in Pakistan. Thus, the trends in childhood obesity highlighted in our study are robust.

Although analysis of the Karachi survey sheds light on the relationship between the level of physical activity and the percentage of children who are overweight and obese, which does seem to track inversely with age, in-depth studies of dietary factors and sociodemographical associates of under- and overnutrition are needed. Finally, a modified International Physical Activity Questionnaire (IPAQ) was used for assessment of physical activity in the Karachi survey. Although this measure is considered adequate for adults, it has been shown to be non-reproducible for all but strenuous physical activity for those aged under 14 years.^40^

In conclusion, the rapidly rising burden of obesity with persistent levels of undernutrition among Indo-Asian children is a unique and complex challenge and represents a major threat to the healthcare services. Thus, there is a clear need to focus health policies on combating this rising epidemic of energy imbalance, which is shifting the pendulum towards overweight and obese children in Indo-Asian countries, while paying attention to the needs of the ones who are still undernourished.

**What is already known on this topic**

- Traditionally, deficiency of macro- and micronutrients has been the major problem among children in low-income countries.

**What this study adds**

- Indo-Asian countries are now experiencing the unique challenge of a rapid rise in childhood obesity despite a persistently high burden of undernutrition.
